# The role of the gut microbiota and probiotics associated with microbial metabolisms in cancer prevention and therapy

**DOI:** 10.3389/fphar.2022.1025860

**Published:** 2022-11-14

**Authors:** Zijun Wang, Lanqing Li, Shunshun Wang, Jing Wei, Linghang Qu, Lianhong Pan, Kang Xu

**Affiliations:** ^1^ Hubei Engineering Technology Research Center of Chinese Materia Medica Processing, College of Pharmacy, Hubei University of Chinese Medicine, Wuhan, China; ^2^ Chongqing Key Laboratory of Development and Utilization of Genuine Medicinal Materials in Three Gorges Reservoir Area, Chongqing Engineering Research Center of Antitumor Natural Drugs, Chongqing Three Gorges Medical College, Chongqing, China

**Keywords:** probiotics, gut microbiota, chemoresistance, oncoloy, immune system

## Abstract

Cancer is the second leading cause of elevated mortality worldwide. Thus, the development of drugs and treatments is needed to enhance the survival rate of the cancer-affected population. Recently, gut microbiota research in the healthy development of the human body has garnered widespread attention. Many reports indicate that changes in the gut microbiota are strongly associated with chronic inflammation-related diseases, including colitis, liver disease, and cancer within the intestine and the extraintestinal tract. Different gut bacteria are vital in the occurrence and development of tumors within the gut and extraintestinal tract. The human gut microbiome has significant implications for human physiology, including metabolism, nutrient absorption, and immune function. Moreover, diet and lifestyle habits are involved in the evolution of the human microbiome throughout the lifetime of the host and are involved in drug metabolism. Probiotics are a functional food with a protective role in cancer development in animal models. Probiotics alter the gut microbiota in the host; thus, beneficial bacterial activity is stimulated, and detrimental activity is inhibited. Clinical applications have revealed that some probiotic strains could reduce the occurrence of postoperative inflammation among cancer patients. An association network was constructed by analyzing the previous literature to explore the role of probiotics from the anti-tumor perspective. Therefore, it provides direction and insights for research on tumor treatment.

## Introduction

Cancer is a deadly malignancy with high clinical significance (Ferlay et al., 2013). It remains one of the leading causes of global death. Over the past century, cancer morbidity and mortality have gradually increased worldwide, mainly new cancer cases in developing countries, from 18.1 million in 2018 to nearly 10 million (or almost one in six) in 2020. Cancers with higher mortality rates are colorectal, liver and breast cancer (Ferlay et al., 2015).

Current clinical treatments for conventional cancer involve surgical, anti-cancer, and radiation therapy. Resection surgery is the primary treatment method, with a high recurrence rate; The adverse drug reactions of anti-cancer drugs are much more severe than those of other drugs, and various side effects, such as vomiting and a decrease in the number of white blood cells, may occur. Moreover, it is difficult to cure them with anti-cancer drugs alone. These chemotherapy drugs and hormones kill cancer cells and damage healthy cells. Drug resistance will develop if the body is given too much of them. The treatment effect will be reduced or disappear (Raguz and Yagüe, 2008). In addition, these cytotoxic drugs pose a significant threat to human health, and these side effects cloud be more severe than the malignant tumors of the cancer. Radiation therapy can be an adjunct to surgery and anti-cancer drugs, with an extended treatment time. Therefore, new treatments with few side effects are urgently required.

Recent research has revealed a specific correlation between daily human life and intestinal flora. Healthy intestinal flora can promote the treatment of tumor patients. While investigating the effect of *Lactobacillus cockerel* on intestinal tumors. Sugimura et al. found that its culture supernatant significantly promoted apoptosis in rectal cancer-like organs. Moreover, *Lactobacillus cockerel* promoted the enrichment of indole-3-lactic acid (ILA), inhibiting intestinal tumorigenesis *in vivo* (Sugimura et al., 2021). Gui et al. studied the effect of gut microbiota on lung cancer mice. They observed that commensal flora contributed to the anti-lung cancer response and that probiotic combination therapy enhanced the anti-growth and anti-apoptotic effects of cisplatin (Gui et al., 2015). Shams et al. analyzed *Candida albicans* isolated from the normal gastrointestinal microbiome of older adults. They demonstrated that *Lactobacillus plantarum* isolated from the gastrointestinal tract and *Candida albicans*, could improve colorectal cancer symptoms (Shams et al., 2021). Growing evidence indicates that the gut microbiota is involved in carcinogenic effects and regulates the activity, efficacy, and toxicity of antineoplastic therapy. These bacteia also target the microbiota to improve efficacy and prevent anti-cancer drug toxicity, behaving as a novel cancer treatment. Probiotics have been shown to induce and lead to changes in physicochemical conditions which further lead to the degradation of carcinogens. Nowak, A et al. studied the probiotic *Lactobacillus casei* DN114001 and determined the ability of the probiotic to metabolize heterocyclic aromatic amines (HCA). These compounds with a high mutagenic potential contribution to the development of colon and gastric cancers, and this probiotic effectively degraded this carcinogen (Nowak and Libudzisz, 2009). Based on the proteomic analysis, Zeng et al. grew *Lactobacillus fermentum* RC42 in NaNO using 0, 100, 300, or 500 mg/L, 8-plex iTRAQ proteomics and bioinformatics analysis to explore the protein expression patterns during nitrite degradation. Therefore, it confirmed the substrate for the role of *Lactobacillus fermentum* RCA in degrading nitrite carcinogens (Zeng et al., 2019).

In this review, the role of probiotics in immuno-tumor and chemotherapy resistance was discussed by taking probiotic supplementation as an adjuvant therapy. We also analyzed the ingested probiotics during chemotherapy to affect drug metabolism, probiotic inhibition of drug resistance, and combination therapy using probiotics.

### The gut microbiota develops and develops with cancer

**FIGURE 1:**
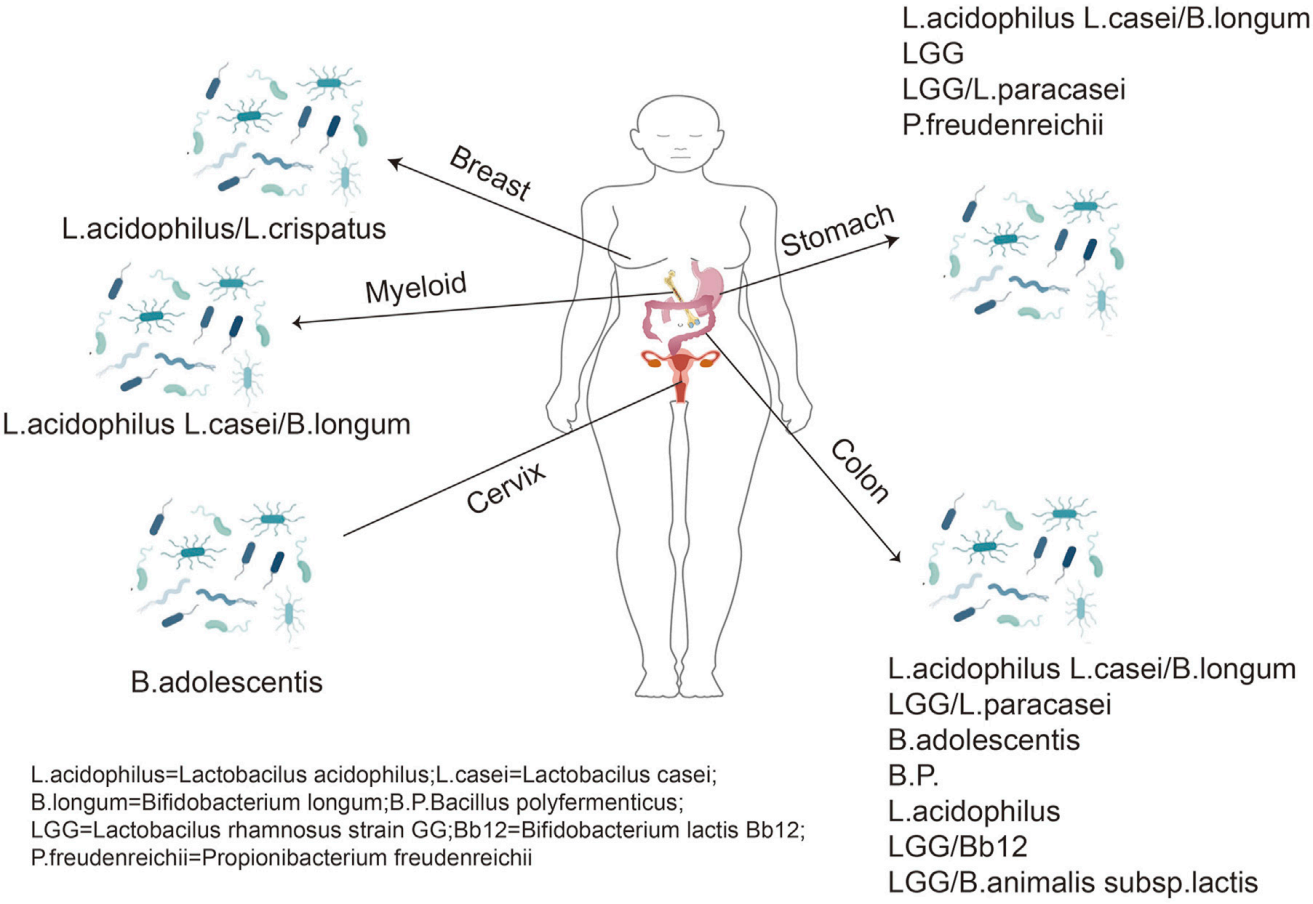
Proliferative effects of probiotics on human cancer cells.

The human gut has tens of billions of organisms (mainly bacteria), representing the most densely populated ecosystem (Eckburg et al., 2005). The gut populations and the genomes of bacteria, fungi, and viruses are called the gut microbiome. The latter encodes genes 150 times more likely than the human genome. Therefore, the gut microenvironment is a complex bioreactor having various biochemical activities (Qin et al., 2010). In equilibrium, the microbiome has a warm and nutrient-rich environment. In contrast, humans benefit from a well-functioning metabolic engine improving our ability to obtain nutrients from food (Maynard et al., 2012). In addition to metabolic aids, the gut microbiome affects tissue development, inflammation, and immunity, thereby promoting human health or producing disease (de Vos and de Vos, 2012; Burcelin et al., 2013). Evidence suggests that the gut microbiota plays a vital role in the occurrence and maintenance of host diseases, such as cancers (Górska et al., 2019). Studies have revealed that destroying intestinal flora impairs the response of cancer cells to platinum complex chemotherapy. It has been demonstrated in mice studies using combination antibiotic therapy (ABX) that gram-negative and gram-positive bacteria and *Lactobacillus fermentum* could affect tumor responses in mice using TLR9 ligands, thus, reducing tumor damage to DNA (Iida et al., 2013). The latest research has indicated the importance of Bifidobacterium for natural anti-tumor immunity and anti-pd-L1 antibody therapy across various tumor settings (Sivan et al., 2015). Therefore, regulating the gut microbiota is reliable in treating diseases of the digestive system, particularly colon cancer (Zheng et al., 2020); Simultaneously, there is a link between gut microbiota and breast cancer (Song et al., 2022). The gut microbiota could be correlated with the development and progression of BC by affecting T cells, neutrophils, and other related inflammatory factors (Yang et al., 2017). In addition, dysbiosis of the gut microbiota can influence the development of hepatocellular carcinoma. Studies have indicated that changes in the composition and function of the gut microbiota are crucial in liver health, from the pre-cirrhotic stage to cirrhosis requirements, decompensation, and liver transplantation (Schwabe et al., 2020). Furthermore, there is an association between dysbiosis of the intestinal flora ecology and different organs (Figure 1).

Among the various strains, probiotics are commonly used food supplements with recognized safety and are fully standardized (O'Toole et al., 2017). Today, probiotics promote food processing and bring health benefits to humans. For instance, *Clostridium butyrate* and the slime-loving bacteria *Ackermannia* can ferment polysaccharides to produce anti-inflammatory SCFAs (Zheng et al., 2020). Additionally, certain strains from *Bacillus* and *Clostridium* can inhibit the multiplication of pathogenic bacteria while protecting intestinal health.

### Functional effects of probiotics

Probiotics are “living microorganisms capable of having a beneficial effect on host health” (Cremon et al., 2018). Probiotics may not be hydrolyzed or absorbed in the upper part of the gastrointestinal tract as a bacterial substrate in the colon. Probiotics are mainly divided into bacterial or lactic acid and non-lactic acid strains and yeasts. *Lactobacillus*, *lactococci*, *bifidobacteria* and *enterococcus* are common bacterial probiotics. Probiotics can alter the gut microbiota and stimulate the activity of bacterial beneficial to the host while inhibiting the activity of bacteria that are detrimental to the host’s health.

Probiotics have several health benefits, such as controlling gastroenteritis, irritable bowel syndrome, liver disease, cardiovascular disease, and immunomodulatory activity (Shida and Nanno, 2008; O'Flaherty et al., 2010). Decreased immune defenses increase the chance of infection and cell carcinogenesis. In constrast, overactive or dysfunctional immune responses can cause inflammatory bowel diseases, allergies, and autoimmune diseases, and probiotics are expected to modulate the immune response to prevent them.

The primary mechanism of action of probiotics, anti-cancer and anti-mutagenic activity is the binding, degradation, and inhibition of probiotics to mutagens. Probiotics prevent and transform harmful, toxic, and highly active carcinogens. SCFAs synthesized during the degradation of indigestible carbohydrates reduce intestinal pH. Moreover, innate immune regulation and enhancement of the host are regulated and promoted by secreting anti-inflammatory molecules (Raman et al., 2013). Probiotic management helps prevent and treat colorectal cancer, and facilitates the safety of traditional cancer treatment management, including chemotherapy, surgery, and radiotherapy (Shamekhi et al., 2020).

Probiotics induce adjuvant effects in specific cellular components (Legesse Bedada et al., 2020). These include cell-mediated regulation of the immune response, activating the reticuloendothelial system, cytokine pathway interleukin and tumor necrosis factor regulation. Lactic acid bacteria can decrease the conversion of pre-carcinogens into carcinogens to obtain colonase levels. They also reduce the level of β-glucosidase, nitro reductase, and azo reductase, directly reducing proto-oncogenes by absorbing nitrites and decreasing secondary bile salt levels (Kumar et al., 2010). AFB1 leads to characteristic genetic changes in the P53 tumor suppressor gene and the RAS proto-oncogene. Thus, they affect the individual risk of hepatocellular carcinoma *L. rhamnosus* can bind AFB1 *in vivo*, thereby preventing AFB1-glutathione binding and decreasing the occurrence of liver cancer (Kumar et al., 2010).

Probiotics can effectively prevent and treat various cancer types. Moreover, the probiotics active cellular material develops multiple beneficial effects in the gastrointestinal tract, releasing different enzymes, including microbial fructosyltransferase (Rawi et al., 2020), pepsin-trypsin, lactose permease, etc. Thus, it creates a potential synergistic effect on digestion (Legesse Bedada et al., 2020). Probiotics are considered safer and more affordable than standard medicines with a long history of use (Choi et al., 2006; Kim et al., 2007). Based on related studies, probiotics and yeasts can eliminate carcinogen toxicity and induce cancer cell death.

## Probiotics can be used as an adjunctive therapy for anti-tumors

The beneficial effect of probiotics depends on the strain utilized, such as *Lactobacillus spp.* And *Bifidobacterium spp* (Lu et al., 2021). The immediate benefits of probiotics include maintaining the gut microbiome balance, reducing potentially pathogenic gastrointestinal microbes, improving gut regularity, and restoring the gut microbiome balance in diarrhea due to antibiotics (Ritchie and Romanuk, 2012). Some studies have revealed that probiotics can also decrease tumor formation and metastasis by regulating microbiota, enhancing intestinal barrier function, and anti-inflammation, in addition to the direct benefits of moderate intake of probiotics on improving host gut microbiota. Chen et al. (2021) fed mice with VSL#3 probiotics, which altered the composition and ratio of the intestinal microbiota. Moreover, it changed the intestinal bacterial composition and altered the abundance of Lactobacillus, Streptococcus, and Lactococcus. It upregulated the concentrations of propionate and butyrate in the intestine and blood. These elevated levels enhanced the expression of chemokine (C-C motif) ligand 20 (CCL20) in lung endothelial cells, leading to the recruitment of T helper 17 (Th17) cells within the lung through the CCL20/chemokine receptor 6 axes. The recruitment of Th17 cells decreased the number of lung tumor foci and attenuated lung metastasis within murine melanoma cells. Blackwood et al. (2017) investigated necrotizing small intestinal colitis (NEC) using *in vivo* and *in vitro* models of *Lactobacillus rhamnosus* and *Lactobacillus plantarum* probiotics. These probiotics were pretreated and cultured using human intestinal Caco-2 cells for *in vitro* experiments. Tight junctions (TJ) were disrupted to mimic NEC, trans-epithelial resistance (TER), and fluorescein isothiocyanate dextran fluxes were determined. TJ structure was assessed using ZO-1 immunofluorescence, yielding that *Lactobacillus* strengthened the intestinal barrier function and preserved TJ integrity.

Studies on many human cancer cells/cell lines have revealed that probiotics have an anti-proliferative or pro-apoptotic activity effect, with colon cancer cells and gastric cancer cells mainly being studied. The latest studies have depicted that the cytoplasmic fractions of *Lactobacillus acidophilus* and *Bifidobacterium longum* indicate significant anti-tumor activity in certain cancer cell lines.

Based on studies of cell lines *in vitro*, probiotics promote cancer cells (Samanta, 2022), Probiotic-based treatment regimens could be used as adjunctive therapies during chemotherapy. Thus, research on the immunomodulatory effects of probiotics on humans is minimal, with a limited number of study subjects. Conversely, quite a few studies showed that taking probiotics (or prebiotics) significantly reduces the incidence of colon cancer in animal models *via* immunomodulatory effects (Drago, 2019). Table 1 shows elevated NK cells or cytotoxicity in rats or mice treated with probiotics. Additionally, probiotics could enhance the immune function in the host *via* the phagocytic activity of macrophages. It has been revealed that probiotics as adjuvant therapeutic agents in chemotherapy can mitigate the deleterious effects of microbiota depletion in patients by enhancing mucus secretion from glandular alveolar cells. It reduces over-activation of NFκB and subsequent release of pro-inflammatory mediators. Moreover, it regulates tight junctions, cross-cellular channels, bacterial translocation, and subsequent immune responses. Thus, it prevents or improves gut flora dysbiosis, and even optimizes the response to chemotherapy (Thomsen et al., 2018).

**TABLE 1:** Immunomodulatory effect of probiotics described in animals or cell lines.

Probiotic products	Subject	Agent	Immune and inflammatory parameters			Ref
			NKcells	Tcells	Macrophages		
A probiotics mixture consisting of *L. acidophilus, L. casei, L. reuteri, B. bifidiu*m, and *Streptococcus thermophilus*	Mouse	2,4,6-Trinitrobenzenesulfonic acid	ND	↑	ND	Kwon et al. (2010)
Bifidobacterium (*B. longum* SP 07/3 and B MF 20/5)	PBMC	None	↑	↑	ND	Dong et al. (2012)
*L. acidophilus*	Mice	None	ND	↑	ND	Urbanska et al. (2016)
*Bacillus subtilis strain* DE111	PBMC	LPS	ND	↑	ND	Freedman et al. (2021)
*L. casei* DN 114001	Mouse	None	ND	↑	↑	Azad et al. (2018)
*Bacillus coagulans*	Mice with colitis	Streptomycin	ND	↑	↑	Bomko et al. (2017)
*Lactobacillus strains*	Human bone marrow dendritic cells	None	ND	↑	ND	Aindelis and Chlichlia, (2020)
*L. plantarum* CJLP243, CJW55-10, and CJLP475	Immunodeficient mice	None	↑	↑	↑	Kang et al. (2022)
*Bifidobacterium strains*	Geriatric patients	None	↑	↑	↑	Akatsu, (2021)
A mixture of *Bifidobacterium, Lactobacillus acidophilus, Lactobacillus casei, Lactobacillus royale* and *Streptococcus thermophilus*	ruminant	None	ND	↑	ND	Raabis et al. (2019)
*L. rhamnosus* GG and *B. lactis* Bb-12	Asthmatic mice	None	ND	↑	ND	Martens et al. (2018)
heat-killed LP8	RAW264.7 cells	LPS	ND	ND	↑	Noh et al. (2022)
LGG	Mouse	UV	ND	↑	ND	Weill et al. (2013)
*L. reuteri*	Mouse	None	ND	↑	ND	Lakritz et al. (2014)
LGG	Caco-2	5-FU	ND	ND	ND	Fang et al. (2014)

ND, no data; PBMC:human peripheral blood mononuclear cells; *L. albicans*, *Lactobacillus albicans*; LGG, *Lactobacillus rhamnosus* GG; *L. plantarum*, *Lactobacillus plantarum*; *L. royale*, *Lactobacillus royale*; *L. plantarum* CJLP243, CJW55-10, and CJLP475, *Lactiplantibacillus plantarum* CJLP243, CJW55-10, and CJLP475; *L. acidophilus*, *Lactobacillus acidophilus*; LP8, *Lactiplantibacillus plantarum* CKDB008; LPS, lipopolysaccharide; 5-FU, 5-fluorouracil.

## Probiotics assist tumor therapy by modulating metabolism

Dysbiosis is an alteration in the composition of the microbiota, disrupting the physiological homeostasis inside the intestinal epithelial cells (Derosa et al., 2018). The causes of biological disorders include dietary changes, antibiotic therapy, and inflammatory bowel disease (Rubinstein et al., 2013). The presaence of dysbiosis may disrupt the mucosal barrier. As a result, the microbiome could influence cancer through various mechanisms, such as producing oncogenic metabolites, involving pro-inflammatory pathways, and suppressing the immune system (Gori et al., 2019). Microbial primary metabolites, such as amino acids, nucleotides, polysaccharides, lipids, and vitamins, help regulate the growth and reproduction of the intestinal flora (Singh et al., 2017). These metabolites are similar across different microbial cells. Synthesizing primary metabolites is a constant process, and any disorder could disrupt normal microbial activity (Yap et al., 2008; Patterson et al., 2014). Microbial secondary metabolites, such as alkaloids, phenols, antibiotics, and pigments, determine the specificity and function of the flora. Tumors have various *in vivo* metabolisms, including amino acid metabolism associated with liver cancer cells (Feng et al., 2021), Glucose metabolism is associated with gastric cancer (Fu et al., 2022). Lipid metabolism is related to liver cancer (Pan et al., 2021) and glycolysis (Li et al., 2022; Pan et al., 2022). Studies have revealed that gut microbes degrade and adsorb nutrients (Rinninella et al., 2019), producting SCFAs (Oliphant and Allen-Vercoe, 2019). Maintaining proper microbiota composition is crucial in preventing pathogens and is integral to the host immune response (Dieterich et al., 2018).

### Carbohydrates

Carbohydrate metabolism is a series of complex chemical reactions inside the human body. The citric acid cycle is the final metabolic pathway and center of the three primary nutrients, carbohydrates, lipids, and amino acids, as the main pathway of carbohydrate metabolism (Narsing Rao et al., 2017; Shi et al., 2017). Carbohydrate metabolism is essential for intestinal flora and colorectal cancer. Anaerobic and aerobic bacteria coexist in the intestine. Superoxides, oxygen radicals, and oxygen molecules are closely associated with the development of colorectal cancer. Various bacteria break down glucose and lactose to synthesize acid (Ribel-Madsen et al., 2016). The intestinal microbial populations are controlled by maintaining acid-base balance and regulating the osmotic pressure.

### Short-chain fatty acids

SCFAs are the primary products of carbohydrate metabolism (Markowiak-Kopeć and Śliżewska, 2020). These include acetic acid, propionic acid, and butyric acid. SCFAs are signaling molecules mediating the interaction between diet, gut microbiota, and host. They have essential roles in the immune, metabolic and endocrine systems within the organism (Boets et al., 2017).

**FIGURE 2:**
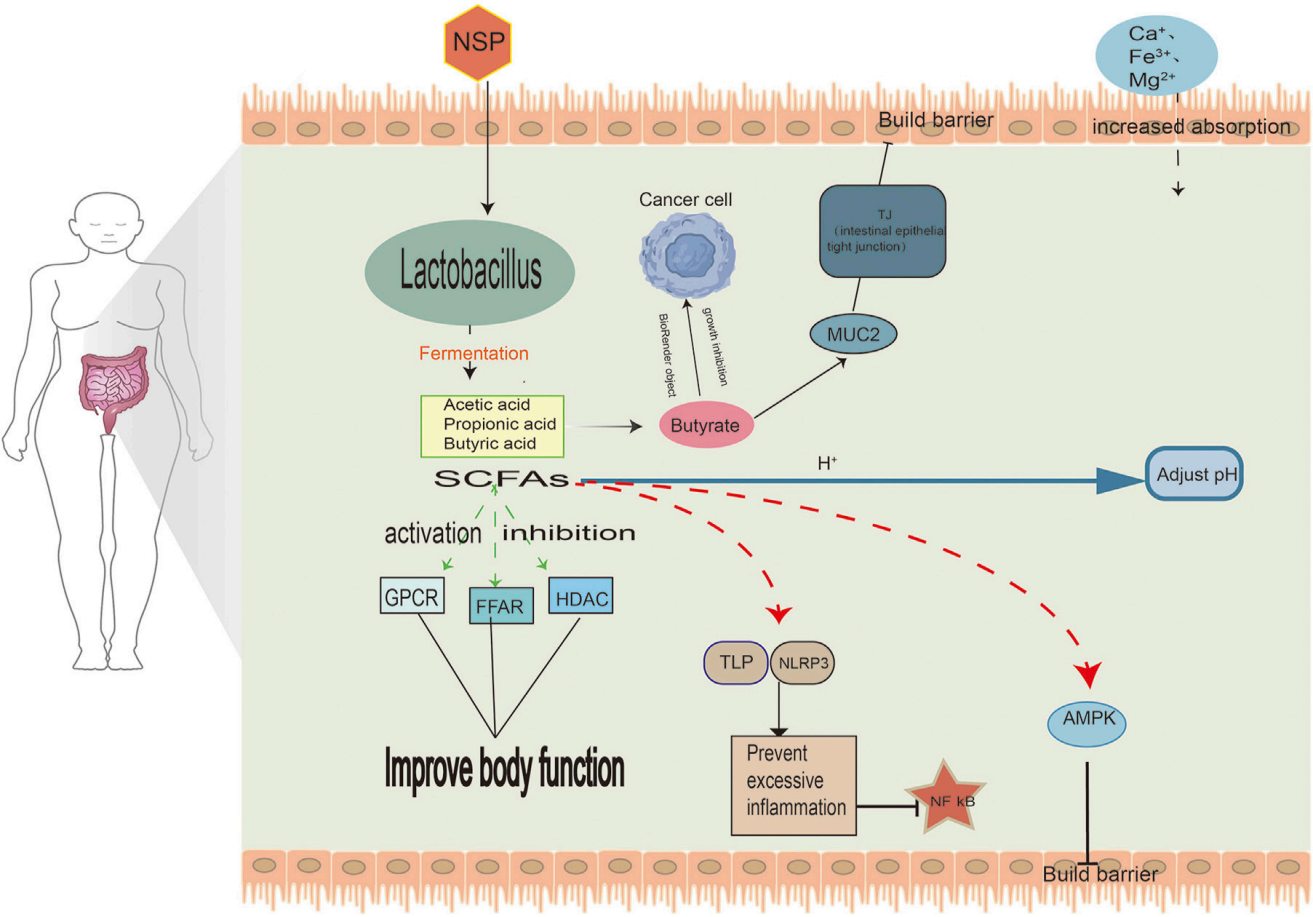
Metabolic effects of SCFAs on the intestinal microbiota.

During the production of SCFA and the increase of butyrate due to the fermentation of wheat grains, maltodextrin inhibits the growth of malignant cells, induces apoptosis, Up-regulation of MUC2 gene expression (Figure 2) and differentiates malignant cells. *Bifidobacterium bifidum* inhibits the growth of cancer cells in CRC by arresting the cell cycle within the G0/G1 phase. Moreover, it increases the activity of alkaline phosphatase, a unique marker whose levels are reduced in malignant cells (Eslami et al., 2019).

### Lipids

Triglycerides, phospholipids, cholesterol, and glycolipids are lipids. Many studies have revealed that a high-fat diet could induce colorectal cancer. A recent study indicated that a high-fat diet could increase bile acid secretion in the colorectum. Some *Clostridium* could accelerate the conversion of bile acids into secondary bile acids by synthesizing various enzymes through fatty acid metabolism (Ridlon et al., 2016; O'Keefe et al., 2015). Secondary bile acids enhance colorectal cancer through different molecular mechanisms as a carcinogen. These include synthesizing oxygen radicals, breaking DNA strands, making chromosomes unstable, and forming cancer stem cells (Ajouz et al., 2014; Farhana et al., 2016). The interactions among fatty acids, bile acids, and intestinal flora can synthesize diacylglycerol, prostaglandins, and leukotrienes, causing tumorigenesis by activating an immune or inflammatory response (Wang and DuBois, 2013; Savari et al., 2014; Kai et al., 2017).

### Tryptophan

Tryptophan is primarily obtained from food with various physiological functions. In humans, tryptophan metabolism occurs mainly in the small intestine and central nervous system and is a crucial regulator of inflammation and immunity. Its metabolites are endogenous ligands of aromatic hydrocarbon receptors (AHR) (Cheng et al., 2015; Islam et al., 2017), regulating the intestinal immune function (Marsland, 2016; Gao et al., 2018). It plays a vital role in mammalian intestinal immune homeostasis.

The latest findings indicate that consuming high tryptophan from the diet and *Lactobacillus reuteri* can enhance the number of immune T cells (Cervantes-Barragan et al., 2017). Predictive experiments on host-microbe interactions have revealed that TNF-α and IFN-γ are related to specific microbial metabolic pathways, including palmitic acid and tryptophan-degrading alcohols (Schirmer et al., 2016). Studies have demonstrated that tryptophan and its metabolites, such as kynulic acid, inhibit inflammation. A specific concentration of tryptophan can significantly inhibit the gene expression of inflammatory factors. Thus, it inhibits disease progression and positively affects human health. Studies have revealed that tumor cells can consume large amounts of tryptophan to synthesize a tumor microenvironmental with an immunosuppressive effect that promotes proliferation. Microorganisms can regulate the metabolism of tryptophan, thus controlling regulating intestinal immunity (Gao et al., 2018).

## Probiotics develop through an immunomodulatory anti-tumor occurrence

It was revealed that the modulation of the structure and composition of the intestinal microbiota after treatment with YINDARA-4 in a rat model of the IBS experiment alleviated visceral hypersensitivity in rats, as well as the normalization of 5-hydroxytryptamine levels in the colon. The decrease in colonic 5-hydroxytryptamine levels after YINDARA-4 treatment may be associated with a decrease in the number of Helicobacter and an enrichment of butyric acid bacteria (Ling et al., 2022).

Studies have depicted that the activity of pro-inflammatory cytokines IFN-γ, TNF-α, and IL-2 levels and NK cells were significantly improved compared with the control group when a group of female mice receiving a subcutaneous injection of breast cancer cells (4T1) was provided with Lactobacillus plantarum rich in selenium nanoparticles. It (SeNPs) resulted in a more effective immune response to cancer (Yazdi et al., 2012). Similarly, oral administration of SeNPs-rich lactic acid bacteria elicited a more effective immune response and decreased liver metastasis in mice breast cancer models. When Linghang et al. (2020) studied leukolides, these ingredients could improve intestinal probiotics in a various ways to alleviate the inflammatory response of colitis and demonstrate better immune effects (Qu et al., 2021; Qu et al., 2022a; Qu et al., 2022b).

Another study indicates that the mechanism of *L. casei* CRL431 on breast cancer lowers IL-6 in serum and mammary glands. The pro-angiogenesis effect of IL-6 is consistent with the inhibitory effect of probiotic supplements against tumor growth. Additionally, the analysis of T lymphocyte subsets revealed that probiotics significantly enhanced the CD8+/CD4+ T lymphocyte proportions. In contrast, there was an increase in CD4^+^ cells in mice that did not receive fermented milk. Thus, probiotics increase the cd8+/CD4+ T lymphocyte ratio since CD4 cells are associated with producing cytokines such as IL-6 and IL-10. Therefore, it will cause IL-6 reduction and delay tumor growth (Aragón et al., 2014; Wang et al., 2020).

First isolated from human milk, Lakritz et al. (2014) reported the protective effect of *Lactobacillus Reuteri* (ATCC-PTA-6475) on tumors in two different mice models of breast cancer. *Lactobacillus Reuteri* was shown to delay the occurrence of tumor features. These included increased incidence of breast tumors, genetically engineered MMTV-HER2/neu, and a high-fat diet feeding. It was a method for constructing models by feeding a high-fat diet to enhance breast cancer independent of ovarian hormones or other risk factors. The present study indicates that modulating the systemic immune cell response to control an increased ratio of CD8+/CD4+ T lymphocytes could effectively counteract dietary or genetic susceptibility to cancer (Subbaramaiah et al., 2011). Based on the mechanism of action, *L. reuteri* protects against cancer by inducing anti-inflammatory CD4^+^ CD25^+^ Treg cells. Thus, it leads to the immune system developing a favorable bias by incorporating systemic treatments against CD25. On the contrary, the protective effect of reuteri leads to breast hyperplasia and an elevation in pretumor lesions (Lakritz et al., 2014). This study explicitly supports the concept that exposure to the lactate microbiota, including those containing the microbiota of L. Reuteri. Therefore, it stimulates Treg cell-guided immune networks and can inhibit inflammatory diseases, such as early malignant transformation. In recent years, it has been shown that tumor control can be achieved through γδ T cell exosomes and the use of chimeric antigen receptor-γδ T cell immunotherapy (Duya et al., 2022).

The synthesis of various studies on the anti-cancer immune response of the above probiotic strains is described in Table 2. Probiotics have a more significant impact on tumor cells. Thus, thoroughly studying various strains and conditions of use can significantly improve the use of probiotics on immunomodulatory anti-tumor and elevate the cancer cure rate.

**TABLE 2:** Effects of probiotic strains on anti-cancer immune response.

Strain	Model	Sample size	Effects on the immune system	Ref
*L.johnsonii and E. hirae*	WT SPF C57BL/6J mice, implanted with MCA205 sarcomas	Three groups of 15 mice were treated with cyclophosphamide	Stimulate the differentiation of CD4^+^ T cells into TH1 and TH17 cells	Subbaramaiah et al. (2011)
*Lactobacillus paracasei* GMNL-133 and *Lactobacillus reuteri* GMNL-89	KC transgenic mice	Four groups were treated with gemcitabine	Increased levels of CD8^+^ T cells and activated CD8^+^ T cells	Chen et al. (2020)
LBB	Carcinogenic male SD rats using DMH	Four groups of ten rats each	Enhanced TLR2	Kuugbee et al. (2016)
*Bacillus coagulans*	A mouse model of immunodeficiency induced by cyclophosphamide *in vivo*	84 mice in 7 groups, cyclophosphamide treatment	IL2 and TNF-a levels decreased	Bomko et al. (2017)
*Lactobacillus plantarum* MH-301	Clinically diagnosed as a patient with locally advanced nasopharyngeal carcinoma	77 patients were randomly assigned (1:1) to receive either a probiotic mixture or placebo	Reversed the upregulation of TLR4/NF-κB	Xia et al. (2021)

LBB, *lactobacillus acidophilus, Bifidobacteria bifidum*, and *Bifidobacteria infantum*; DMH, 1,2-dimethylhydrazine dihydrochloride.

## Anti-tumor combination therapy with probiotics

Recent years have seen a growing interest in using probiotics combined with conventional cancer treatments. In 1993, a comparative study of 223 patients showed that combination therapy, such as radiation therapy and treatment using the *Lactobacillus casei* strain (LC9018), enhanced the induction of immune response mechanisms to cancer cells. Thus, it improved tumor regression in cervical cancer patients (Okawa et al., 1993). Studies of azomethane-induced mice models of CRC treated using a probiotic mixture of seven strains of *Lactobacillus*, *Bifidobacterium* and *Streptococcus* have revealed that colon cancer is inhibited by the changes in modulating MUCOS CD4^+^ T polarization and overall gene expression (Bassaganya-Riera et al., 2012).

Probiotic supplement therapy possesses broad application prospects to enhance the effect of antibiotics. It can achieve better therapeutic outcomes and maintain the balance of the host gastrointestinal microbiota. Fonseca *et al.* analyzed 10 clinical trials undergoing antibiotic therapy supplementation using different strains of *Lactobacillus reuteri*. Except for one study, it showed more significant eradication effects than the single antibiotic group (Feng et al., 2017). Sýkora et al. (2005) *.* treated 86 children having *Helicobacter pylori* infection. They found that the eradication rate of DN114001 using Caseinella was 84.6% along with triple therapy, compared with 57.5% in the triple therapy group alone. Probiotic supplementation simultaneously decreased the severity of side effects. Probiotic supplementation therapy can improve the eradication rate of antibiotic-sensitive strains of *Helicobacter pylori* and the eradication rate of resistant strains. For clarithromycin-resistant strains, *Lactobacillus lactobacillus* OLL2716 plus triple therapy enhanced the eradication rates by approximately 10% than the single triple therapy (Deguchi et al., 2012). In addition, fermented milk preparations with a variety of probiotics can also enhance the eradication rate of triple therapy by 5%–15% (Sachdeva and Nagpal, 2009).

Experiments using animal models and clinical trials have revealed that probiotics positively affect the host gastrointestinal microbiota. After treatment using *Lactobacillus rhamnosus* GCNL-74 or *Lactobacillus acidophilus* GCNL-185, the abundance of *Helicobacter pylori* infection in mice having *Bifidobacterium spp*. And Ackermann’s mucosal parents elevated significantly (Chen et al., 2019). Wu *et al.* observed that gut microbiota diversity decreased significantly during supplementation using *Bacillus subtilis* and fecal E when *Helicobacter pylori*-infected individuals were treated using triple therapy alone. Moreover, colonizing the stomach using specific probiotics can regulate the balance of the gastric microbiota (Espinoza et al., 2018). Therefore, probiotic supplements can maintain the gastrointestinal and intestinal flora during *Helicobacter pylori* infection and antibiotic therapy.

## Probiotics inhibit anti-tumor resistance

Probiotics possess many beneficial properties to control pathogenic bacteria, such as improving intestinal barrier function, competitive rejection by reducing adhesion to cells, and producing organic acids that antagonize disease-causing bacteria. Many probiotics have antibacterial compounds such as short-chain fatty acids, hydrogen peroxide, nitric oxide, and bacteriocins. It enhances their ability to compete against other gastrointestinal microbes, thereby inhibiting pathogenic bacteria (Atassi and Servin, 2010; Chenoll et al., 2011).

Certain probiotics can colonize the nervous regions of the stomach, while most probiotics are localized in the gut (Francavilla et al., 2008; Ryan et al., 2008). Substances with the metabolism of these strains could have the potential to work synergistically with antibiotics. Yang and Yang, 2018 performed antibacterial studies on *Clostridium difficile*. The results indicated that *Bifidobacterium breve* YH68 cell-free supernatant promoted the synergistic effect of antibiotics and weakened antagonism. Thus, combining probiotic metabolites and antibiotics can have a more significant antibacterial effect on Gram-negative or Gram-positive pathogens. While some *in vitro* studies on *Helicobacter pylori* confirmed the synergy between probiotics and antibiotics, positive clinical results describe potential interactions.

Emara et al. (2016) focused on probiotics from a histopathological perspective. They found that probiotics decreased the density of *Helicobacter pylori* on the epithelial luminal side, leading to long-term improvements in histological inflammation and activity score time inside the stomach and sinuses. This can be useful in treating gastric malignancies, such as mucosa-associated lymphoid tissue lymphoma and adenocarcinoma. Simultaneously, the eradication of *Helicobacter pylori* affects the intestinal microbiota (Jakobsson et al., 2010; Malfertheiner et al., 2017). Adding Pbs to standard triple therapy (e.g., *Streptococcus faecalis* and *Bacillus subtilis*) could limit the growth of antibiotic-resistant bacteria and reduce fluctuations within the gut microbiota (Oh et al., 2016). Probiotics have been revealed to restrict tumor growth in homozygous mice models of colon cancer and NRAS-mutant melanoma. Moreover, it enhances the efficacy of MEK inhibitors against melanoma while delaying drug resistance (Li et al., 2020).

## Conclusion

The gut microbiota has excellent potential for therapeutic capabilities against cancer. The medical significance of probiotics as bacterial substrates within the colon and their beneficial effects on host health is advancing. Although only some probiotics currently show anti-cancer properties, the function of other probiotics should be studied. Probiotics as an adjunctive treatment are potentially beneficial in cancer prevention and treatment. It positively affects the metabolism of the gut microbiota during chemotherapy, thereby decreasing its toxic side effects. Currently, the probiotic treatment of cancer mainly uses combination therapy, including radiotherapy and heat-killing *Lactobacillus casei* strain (LC9018) therapy. Other therapies are waiting to be explored. The research on probiotic inhibition resistance is primarily observed in probiotic inhibition of antibiotic treatment *Helicobacter pylori*. However, few studies on probiotics indicate inhibitory resistance in other aspects. Despite this, probiotics still have significant potential in cancer treatment and tumor immunity.

## Abbreviations

SCFAs, Short-chain fatty acids; ND, no data; PBMC: Human peripheral blood mononuclear cells; L. albicans, Lactobacillus albicans; LGG, Lactobacillus rhamnosus GG; L. plantarum, Lactobacillus plantarum; L. royale, Lactobacillus royale; L. plantarum CJLP243, CJW55-10, and CJLP475 = Lactiplantibacillus plantarum CJLP243, CJW55-10, and CJLP475; L. acidophilus, Lactobacillus acidophilus; LP8, Lactiplantibacillus plantarum CKDB008; LPS, lipopolysaccharide; 5-FU, 5-fluorouracil; LBB, Lactobacillus acidophilus, Bifidobacteria bifidum, and Bifidobacteria infantum; DMH, 1,2-dimethylhydrazine dihydrochloride.
